# Prognostic and Clinicopathological Significance of PD-L1 in Patients with Cholangiocarcinoma: A Meta-Analysis

**DOI:** 10.1155/2020/1817931

**Published:** 2020-07-15

**Authors:** Qinfen Xie, Lidong Wang, Shusen Zheng

**Affiliations:** ^1^Zhejiang University School of Medicine, Hangzhou 310058, China; ^2^Department of Hepatobiliary and Pancreatic Surgery, Shulan (Hangzhou) Hospital Affiliated to Zhejiang Shuren University Shulan International Medical College, Hangzhou 310000, China

## Abstract

**Background:**

In recent years, there is growing literature on the prognostic significance of programmed death-ligand 1 (PD-L1) in cholangiocarcinoma (CCA); however, data have been conflicting. Therefore, the objective of this study was to assess the correlation between PD-L1 and prognosis in CCA through meta-analysis.

**Methods:**

Published studies were retrieved from the Web of Science, PubMed, Embase, and Cochrane Library up to April 17, 2020. The relationships between PD-L1 expression and survival outcomes were assessed using hazard ratios (HRs) and 95% confidence intervals (CIs).

**Results:**

Eighteen studies consisting of 2012 patients were included. Overexpression of PD-L1 was significantly associated with worse overall survival (OS) (HR = 1.58, 95%CI = 1.30 − 1.92, *p* < 0.001) but not with poor disease-free survival (DFS) (HR = 1.03, 95%CI = 0.68 − 1.55, *p* = 0.895) in CCA. Moreover, PD-L1 was associated with low differentiation (OR = 1.43, 95%CI = 1.09 − 1.87, *p* = 0.010) and higher pN stage (OR = 1.45, 95%CI = 1.10 − 1.92, *p* = 0.009) but not with sex, TNM stage, vascular invasion, perineural invasion, age, or tumor size.

**Conclusion:**

High PD-L1 expression was associated with worse OS, poor differentiation, and higher pN stage in patients with CCA. PD-L1 could be a potential prognostic marker in CCA.

## 1. Introduction

Cholangiocarcinoma (CCA) is the second most frequent type of primary liver cancer, with aggressive nature and a high mortality rate, accounting for 20% of liver-related deaths [1]. The incidence of CCA is increasing during the past decades in Western countries, and the 5-year survival rate is approximately 10% [2, 3]. Surgical resection is the definitive treatment option for CCA; however, recurrence remains high and maintains a poor prognosis [4, 5]. Emerging treatment options, including targeted therapies and immunotherapy with checkpoint inhibitors, are in clinical trials and provide personalized therapeutic strategies for patients with CCA [5]. Efficient prognostic biomarkers are still lacking for CCA; therefore, a reliable prognostic marker is needed for optimal therapeutic strategy selection [6].

In recent years, the tumor microenvironment and immune milieu have attracted much attention [7]. The immune checkpoint molecules, programmed cell death-1 (PD-1) and its ligand programmed death-ligand-1 (PD-L1), regulate immune responses in cancer development [8]. Activation of the PD-1/PD-L1 axis results in immune suppression by inhibition of immune cells and secretion of certain cytokines [9]. Recent evidence also showed the prognostic value of PD-L1 in different types of cancers [10]. The prognostic role of PD-L1 in CCA has also been investigated; however, data were inconsistent [11–28]. Therefore, we conducted a meta-analysis to explore the prognostic and clinicopathologic roles of PD-L1 in patients with CCA.

## 2. Materials and Methods

This meta-analysis was conducted based on the Preferred Reporting Items for Systematic Reviews and Meta-Analyses statement [29]. Ethical approval and patient consent were not performed because all data collected were from previously published studies.

### 2.1. Literature Search

PubMed, Web of Science, Cochrane Library, and Embase were reviewed till April 17, 2020. The search terms used were “PD-L1” or “programmed death ligand 1” or “PDL1” or “B7-H1” or “CD274”, and “bile duct neoplasms” or “cholangiocarcinoma” or “bile duct cancer”. The reference lists in relevant studies were also examined for potential inclusions.

### 2.2. Inclusion and Exclusion Criteria

The criteria for inclusion were (1) patients histologically diagnosed with CCA; (2) PD-L1 expression detected by immunohistochemistry (IHC); (3) studies reporting the relationship between PD-L1 and survival outcomes including overall survival (OS) and disease-free survival (DFS); (4) sufficient data available for the calculation of hazard ratios (HRs), odds ratios (ORs), and 95% confidence intervals (CIs); and (5) studies published in English.

The exclusion criteria were (1) conference abstracts, case reports, reviews, or letters; (2) studies with insufficient data for analysis; (3) animal studies; and (4) studies recruited overlapping patients.

### 2.3. Data Extraction

Two independent investigators (Q.X. and L.W.) collected data from the included studies and any discrepancies were settled by discussion with a senior investigator (S.Z.). The following baseline information was extracted: author, year, study country, study design, sample size, treatment method, follow-up, survival outcomes, positive rate of PD-L1 expression, and detection method. Detailed information on PD-L1 antibodies used for IHC (specie, clone, dilution, source, and cutoff value) was also extracted. The HR and 95% CIs of OS and DFS were obtained directly if reported or were calculated by Tierney's method [30].

### 2.4. Quality Assessment

The Newcastle-Ottawa Scale (NOS) was applied to evaluate the quality of eligible studies [31]. The NOS evaluated each study in three aspects. The score ranges from 0-9, and studies with NOS scores of ≥6 are considered high-quality studies.

### 2.5. Statistical Analysis

The relationships between PD-L1, OS, and DFS were assessed by combining HRs and 95% CIs. Chi-squared tests and inconsistency index (*I*^2^) statistics were used to examine heterogeneity. In the presence of significant heterogeneity (*I*^2^ > 50%), a random-effect (REM) model was used; otherwise, a fixed-effect model (FEM) was applied. ORs and 95% CIs were used as effective sizes to assess the association between PD-L1 and clinicopathological features. Publication bias was tested using Begg's and Egger's tests. A *p* < 0.05 was considered to be statistically significant. All statistical analyses were conducted using Stata version 12.0 (StataCorp. 2011. Stata Statistical Software: Release 12. College Station, TX: StataCorp LP.).

## 3. Results

### 3.1. Study Characteristics

**Figure 1:**
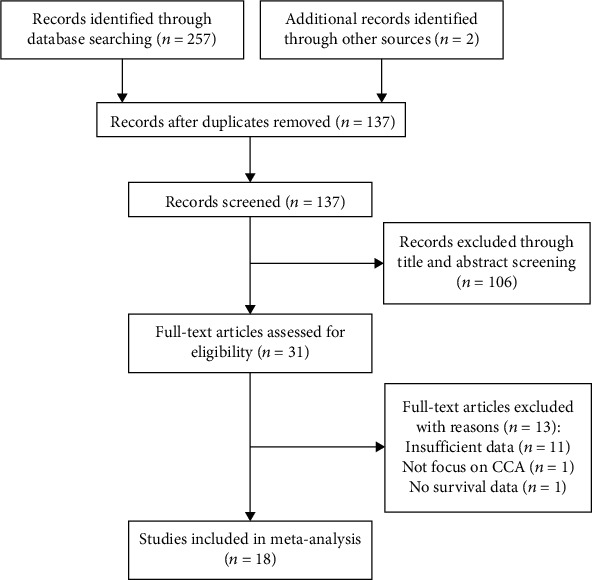
Flow chart of literature search and study selection.

The initial literature search identified 259 studies. According to the selection criteria, a total of 18 studies [11–28] with 2012 patients were eventually included in the meta-analysis (Figure 1). The basic characteristics of the eligible studies are shown in Table 1. Seven studies were conducted in China [13, 15, 16, 21, 22, 27, 28], three in Japan [18, 24, 25], two in Korea [11, 20], two in Germany [19, 26], two in Germany [14, 17], and one in Thailand [23] and UK [12]. One study was of prospective design [12], and 17 were retrospective cohort studies [11, 13–28]. The sample size ranged from 26 to 320. All included studies had a NOS score of ≥6. Detailed information on the primary antibody used for PD-L1 is summarized in Table 2. All included studies used IHC to detect PD-L1 expression. The cutoff values to stratify high- and low expression of PD-L1 were different, including 1%, 5%, H-score 5, score 3, and 2+.

### 3.2. Prognostic Value of PD-L1 in OS, DFS, and Subgroup Analysis

**Figure 2:**
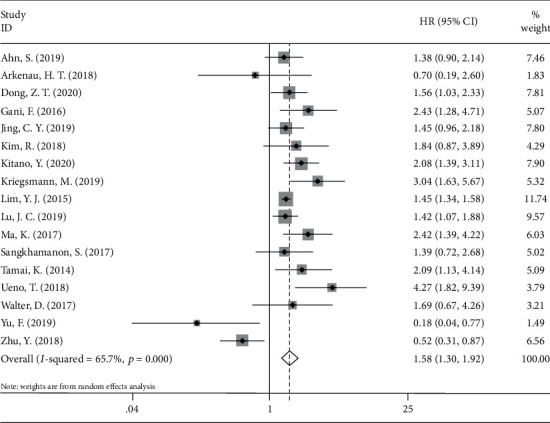
Forest plots for the association between PD-L1 expression and overall survival.

**Figure 3:**
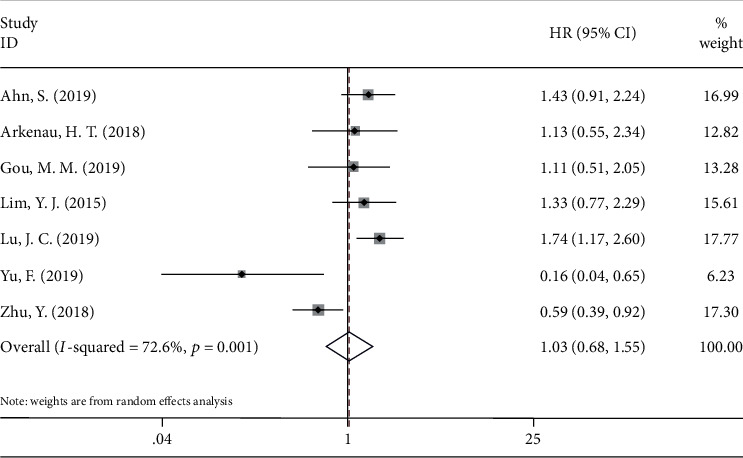
Forest plots for the association between PD-L1 expression and disease-free survival.

Seventeen studies [11–14, 16–28] with a total of 1982 patients reported a correlation between PD-L1 and OS. The pooled HR and 95% CI suggested that overexpression of PD-L1 was significantly correlated with worse OS (HR = 1.58, 95%CI = 1.30 − 1.92, p < 0.001) in patients with CCA (Figure 2; Table 3). For DFS, a total of 7 studies [11, 12, 15, 20, 21, 27, 28] with 896 patients provided the relevant data. The forest plot (Figure 3) showed that PD-L1 expression was not significantly correlated with DFS (HR = 1.03, 95%CI = 0.68 − 1.55, *p* = 0.895) (Table 3). To further investigate the source of heterogeneity, subgroup analysis stratified by ethnicity (Caucasian and Asian), tumor location (iCCA, eCCA, and CCA), and treatment (surgery and nonsurgery) was performed for OS and DFS. For OS, PD-L1 overexpression remained a prognostic factor for patients of Caucasian (HR = 2.14, 95%CI = 1.52 − 3.02, *p* < 0.001) and Asian (HR = 1.49, 95%CI = 1.20 − 1.84, *p* < 0.001) ethnicity; and for those receiving surgery (HR = 1.61, 95%CI = 1.32 − 1.95, *p* < 0.001) (Table 3). Notably, regarding tumor location, high PD-L1 expression was a prognostic factor for patients with eCCA (HR = 1.71, 95%CI = 1.25 − 2.36, *p* < 0.001) and CCA including iCCA and eCCA (HR = 1.98, 95%CI = 1.47 − 2.65, *p* < 0.001). However, elevated PD-L1 expression did not correlate with worse OS in patients with iCCA (HR = 1.31, 95%CI = 0.88 − 1.95, *p* = 0.180) (Table 3). For DFS, the subgroup analysis indicated that PD-L1 overexpression had no significant prognostic value regardless of ethnicity, treatment method, or tumor location (Table 3).

### 3.3. PD-L1 and Clinicopathological Characteristics of CCA

**Figure 4:**
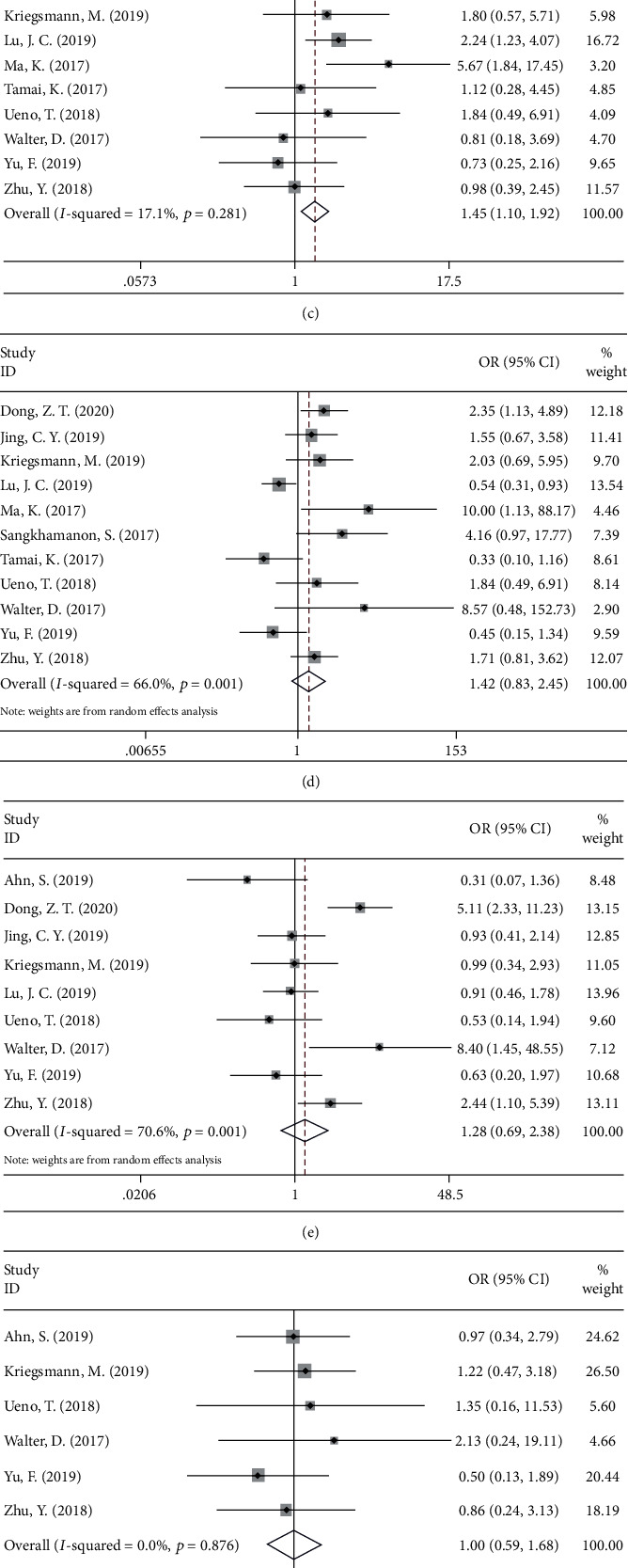
Forest plots of ORs for the association between PD-L1 expression and (a) sex (male vs. female), (b) tumor differentiation (poor vs. well/moderate), (c) pN stage (III+IV vs. I+II), (d) TNM stage (III+IV vs. I+II), (e) vascular invasion (yes vs. no), (f) perineural invasion (yes vs. no), (g) age (>60 vs. ≤60), and (h) tumor size (>5 cm vs. ≤5 cm).

Thirteen studies [11, 13, 16, 18, 19, 21–28] investigated the relationship between PD-L1 and the following eight clinicopathological factors: sex (male vs. female), tumor differentiation (poor vs. well/moderate), pN stage (III+IV vs. I+II), TNM stage (III+IV vs. I+II), vascular invasion (yes vs. no), perineural invasion (yes vs. no), age (>60 vs. ≤60), and tumor size (>5 cm vs. ≤5 cm). As shown in Figure 4, high PD-L1 expression was correlated with poor differentiation (OR = 1.43, 95%CI = 1.09 − 1.87, *p* = 0.010) and higher pN stage (OR = 1.45, 95%CI = 1.10 − 1.92, *p* = 0.009). However, no significant correlation was found between PD-L1 and sex (OR = 1.23, 95%CI = 0.95 − 1.58, *p* = 0.114), TNM stage (OR = 1.42, 95%CI = 0.83 − 2.45, *p* = 0.204), vascular invasion (OR = 1.28, 95%CI = 0.69 − 2.38, *p* = 0.431), perineural invasion (OR = 1.00, 95%CI = 0.59 − 1.68, *p* = 0.994), age (OR = 0.90, 95%CI = 0.61 − 1.33, *p* = 0.609), and tumor size (OR = 0.97, 95%CI = 0.70 − 1.33, *p* = 0.828).

### 3.4. Publication Bias

Begg's funnel plot and Egger's tests were applied to evaluate the publication bias in this meta-analysis. There was no obvious publication bias for OS (Begg's test of *p* = 0.902, Egger's test of *p* = 0.670) or DFS (Begg's test of *p* = 0.230, Egger's test of *p* = 0.266).

## 4. Discussion

CCA is an aggressive cancer, and most patients present at an advanced stage at the time of diagnosis [32, 33]. The current meta-analysis containing 18 studies with 2012 patients showed that high PD-L1 expression was a significant prognostic factor for low OS (HR = 1.58). Particularly, the mortality risk of patients with CCA with high PD-L1 expression increased by 58% compared with that of patients with low PD-L1 expression. PD-L1 expression was not significantly correlated with DFS. In addition, we found that PD-L1 was positively associated with poor differentiation and higher pN stage in CCA. Generally, these results demonstrated that PD-L1 overexpression was associated with invasive clinical features and suggested poorer prognosis of CCA.

The tumor microenvironment in CCA consists of cancer cells, stromal cells, and various immune cells including CCA cells, cancer-associated fibroblasts, tumor-associated macrophages, tumor-infiltrating lymphocytes, and CD8+ cytotoxic T lymphocytes [34]. PD-1 is expressed on B cells, activated CD4+ and CD8+ T cells, and dendritic cells [35]. PD-L1 is a ligand of PD-1 and is expressed on different cell types [36]. Targeting PD-1/PD-L1 is a new strategy for cancer immunotherapy [37]. Recent studies showed that nivolumab (a PD-1 inhibitor) showed considerable safety in patients with metastatic CCA [15]. PD-L1 is mainly expressed by intertumoral immune cells in CCA [38]. Thus, the overexpression of PD-L1 may lead to immune tolerance in the tumor environment and result in tumor progression. This could be a possible mechanism for the correlation between PD-L1 elevation and poor differentiation in CCA.

Recent studies have demonstrated that PD-L1 overexpression is associated with unfavorable prognosis in various types of cancer [39, 40]. A recent meta-analysis showed that high expression of PD-L1 was significantly associated with a poor OS (HR = 1.22, 95%CI = 1.01 − 1.48, *p* = 0.04) in colorectal cancer [41]. Another meta-analysis including 13 studies also demonstrated that tumor cell PD-L1 expression was correlated with poor OS (HR = 2.128, 95%CI : 1.341–3.378, *p* = 0.001) in patients with diffuse large B-cell lymphoma [42]. These findings were consistent with the results of this study. Notably, in the current meta-analysis, we included studies using the IHC method to detect PD-L1. The antibodies used for PD-L1 and cutoff values vary among the included studies, which may result in heterogeneity. However, we failed to observe a significant prognostic role of PD-L1 in DFS in patients with CCA. This negative result may be due to the limited sample size, wherein only 7 studies with 896 patients were included for DFS analysis.

Notably, several limitations of this study should be acknowledged. First, the cutoff values of PD-L1 varied in the included studies (Table 2), which may introduce heterogeneity. Further investigations used uniform antibody and a cut-off value of PD-L1 are needed. Second, only one included study was a prospective trial, and the remaining were retrospective studies. Therefore, high-quality prospective studies are still needed. Third, some HRs and 95% CIs were calculated according to survival curves, which may not be as precise as the original data. Fourth, the sample was relatively small. Only 2012 patients were enrolled and most patients were of Asian ethnicity. More studies recruiting patients of diverse ethnicities were needed to verify the results of this meta-analysis. Because of these limitations, well-designed large cohort studies or randomized controlled trials may be recommended to confirm our findings.

## 5. Conclusions

Our study indicates that PD-L1 was associated with worse OS, poor differentiation, and higher pN stage in patients with CCA. PD-L1 could be a potential prognostic marker for CCA.

## Figures and Tables

**Table 1:** Characteristics of the studies included in the meta-analysis.

Author	Year	Country	No. of patients	Ethnicity	Sex (M/F)	Tumor location	Follow-up (months) Median (range)	Study design	Treatment	Survival outcome	PD-L1 (+) *n* (%)	NOS score
Ahn, S.	2019	Korea	183	Asian	122/61	eCCA	27.2	Retrospective	Surgery	OS, DFS	31 (16.9)	7
Arkenau, H. T.	2018	UK	26	Caucasian	8/18	CCA	6.4 (4.1-13.2)	Prospective	Targeted therapy + immunotherapy	OS, DFS	12 (46.2)	8
Dong, Z. T.	2020	China	125	Asian	58/77	iCCA	16 (5-63)	Retrospective	Surgery	OS, DFS	52 (41.6)	7
Gani, F.	2016	USA	54	Caucasian	17/37	iCCA	NR	Retrospective	Surgery	OS	39 (72.2)	6
Gou, M. M.	2019	China	30	Asian	18/12	CCA	To Sep 2018	Retrospective	Immunotherapy	DFS	11 (36.6)	7
Jing, C. Y.	2019	China	153	Asian	NR	iCCA	47.5 (1-88.4)	Retrospective	Surgery	OS	43 (28.1)	7
Kim, R.	2018	USA	44	Caucasian	23/21	eCCA	NR	Retrospective	Surgery	OS	10 (22.7)	6
Kitano, Y.	2020	Japan	177	Asian	115/62	CCA	78.7	Retrospective	Surgery	OS	54 (30.5)	6
Kriegsmann, M.	2019	Germany	170	Caucasian	109/61	CCA	NR	Retrospective	Surgery	OS	19 (11.1)	6
Lim, Y. J.	2015	Korea	83	Asian	61/22	eCCA	27	Retrospective	Surgery	OS, DFS	56 (83)	7
Lu, J. C.	2019	China	320	Asian	19/129	iCCA	To Oct 2016	Retrospective	Surgery	OS, DFS	99 (30.9)	7
Ma, K.	2017	China	70	Asian	38/32	eCCA	To Mar 2015	Retrospective	Surgery	OS	30 (42.9)	7
Sangkhamanon, S.	2017	Thailand	46	Asian	33/13	CCA	NR	Retrospective	Surgery	OS	18 (39.1)	6
Tamai, K.	2014	Japan	91	Asian	62/29	eCCA	NR	Retrospective	Surgery	OS	77 (84.6)	6
Ueno, T.	2018	Japan	117	Asian	93/24	eCCA	27 (0-189)	Retrospective	Surgery	OS	10 (8.5)	7
Walter, D.	2017	Germany	69	Caucasian	50/19	eCCA	23 (0-100)	Retrospective	Surgery	OS	8 (11.6)	7
Yu, F.	2019	China	62	Asian	41/21	eCCA	NR	Retrospective	Surgery	OS, DFS	20 (32.3)	6
Zhu, Y.	2018	China	192	Asian	115/77	iCCA	24 (0.4-85)	Retrospective	Surgery	OS, DFS	34 (17.7)	7

CCA: cholangiocarcinoma; eCCA: extrahepatic cholangiocarcinoma; iCCA: intrahepatic cholangiocarcinoma; NR: not reported; OS: overall survival; DFS: disease-free survival; NOS: Newcastle-Ottawa scale. CCA includes iCCA and eCCA.

**Table 2:** Immunohistochemical technique used in the studies included in the meta-analysis.

Author	Year	Detection method	Primary antibody				Source	Cut-off value
			Antibody	Specie	Clone	Dilution		
Ahn, S.	2019	IHC	Anti-PD-L1	Mouse, MAB	22C3	1 : 100	Dako, Carpinteria, CA, USA	1%
Arkenau, H. T.	2018	IHC	Anti-PD-L1	Mouse, MAB	22C3	NR	Agilent, Carpinteria, CA	1%
Dong, Z. T.	2020	IHC	Anti-PD-L1	MAB	NR	NR	Cell Signaling Technology, Inc. Danvers, MA, USA	5%
Gani, F.	2016	IHC	Anti-PD-L1	Mouse, MAB	5H1	NR	NR	5%
Gou, M. M.	2019	IHC	Anti-PD-L1	NR	NR	NR	NR	1%
Jing, C. Y.	2019	IHC	Anti-PD-L1	Rabbit, MAB	E1L3N	1 : 200	Cell Signaling Technology, MA, USA	5%
Kim, R.	2018	IHC	Anti-PD-L1	Mouse, MAB	5H1	NR	NR	1%
Kitano, Y.	2020	IHC	Anti-PD-L1	Rabbit, MAB	E1L3N	1 : 200	Cell Signaling Technology, Tokyo, Japan	5%
Kriegsmann, M.	2019	IHC	Anti-PD-L1	NR	SP263	NR	Roche AG, Rotkreuz, Switzerland	1%
Lim, Y. J.	2015	IHC	Anti-PD-L1	Rabbit, MAB	E1L3N	1 : 100	Cell Signaling Technology, Danvers, MA, USA	H-score 5
Lu, J. C.	2019	IHC	Anti-PD-L1	Rabbit, MAB	SP142	1 : 100	GeneTech Co. Ltd., Shanghai, China	5%
Ma, K.	2017	IHC	Anti-PD-L1	Rabbit, MAB	NR	1 : 250	Abcam, Cambridge, MA, USA	5%
Sangkhamanon, S.	2017	IHC	Anti-PD-L1	NR	NR	1 : 1000	Roche Diagnostic GmbH, USA	1%
Tamai, K.	2014	IHC	Anti-CD274	Rabbit, PAB	NR	NR	Abcam, Cambridge, MA, USA	++
Ueno, T.	2018	IHC	Anti-PD-L1	NR	NR	NR	NR	5%
Walter, D.	2017	IHC	Anti-PD-L1	Rabbit, MAB	E1L3N	1 : 50	Cell Signaling Technology, Danvers, MA, USA	Score 3
Yu, F.	2019	IHC	Anti-PD-L1	Rabbit, MAB	E1L3N	1 : 200	Cell Signaling Technology, Danvers, MA, USA	Score 3
Zhu, Y.	2018	IHC	Anti-PD-L1	Rabbit, MAB	SP142	1 : 50	Spring Bioscience, Inc., CA, USA	5%

MAB: monoclonal antibody; IHC: immunohistochemistry; NR: not reported; PAB: polyclonal antibody.

**Table 3:** Subgroup analysis of the prognostic value of PD-L1 in OS and DFS in CCA.

Subgroup factors	No. of studies	No. of patients	HR (95% CI)	*p*	Effects model	Heterogeneity	
						*I* ^2^ (%)	*p*
Overall survival							
Total	17	1982	1.58 (1.30-1.92)	<0.001	REM	65.7	<0.001
Ethnicity							
Caucasian	5	363	2.14 (1.52-3.02)	<0.001	FEM	11.8	0.338
Asian	12	1619	1.49 (1.20-1.84)	<0.001	REM	70.7	<0.001
Treatment							
Surgery	16	1956	1.61 (1.32-1.95)	<0.001	REM	67.0	<0.001
Nonsurgery	1	26	0.70 (0.19-2.60)	0.595	—	—	—
Tumor location							
iCCA	5	844	1.31 (0.88-1.95)	0.180	REM	76.9	0.002
eCCA	8	719	1.71 (1.25-2.36)	<0.001	REM	63.1	0.008
CCA	4	419	1.98 (1.47-2.65)	<0.001	FEM	44.2	0.146
Disease-free survival							
Total	7	896	1.03 (0.68-1.55)	0.895	REM	72.6	0.001
Ethnicity							
Caucasian	1	26	1.13 (0.55-2.34)	0.742	—	—	—
Asian	6	870	1.00 (0.62-1.61)	0.991	REM	77.2	0.001
Treatment							
Surgery	5	840	0.97 (0.56-1.69)	0.911	REM	81.7	<0.001
Non-surgery	2	56	1.12 (0.67-1.85)	0.667	FEM	0	0.965
Tumor location							
iCCA	2	512	1.02 (0.36-2.92)	0.972	REM	92.2	<0.001
eCCA	3	328	0. (0.40-2.03)	0.800	REM	77.1	0.013
CCA	2	56	1.12 (0.67-1.85)	0.667	FEM	0	0.965

FEM: fixed-effects model; REM: random-effects model.

## Data Availability

The data used to support the findings of this study are available from the corresponding author upon request.
